# Docetaxel-Induced Stevens-Johnson Syndrome in a Patient with Metastatic Prostate Adenocarcinoma

**DOI:** 10.1155/2019/7928752

**Published:** 2019-01-08

**Authors:** Osama Diab, Dan Mcentire, Thamer Kassim, Ali Nayfeh, Abdel Rahman Dajani, Mitchell Kerfeld, Jonathon Campbell, Adbullah Alsuwaidan, Mahmoud Abu Hazeem, Maryam Gbadamosi-Akindele

**Affiliations:** ^1^Department of Medicine, Creighton University, Omaha, NE, USA; ^2^Department of Medicine, University of Utah, Salt Lake City, UT, USA; ^3^Department of Medicine, Norwalk Hospital, Norwalk, CT, USA; ^4^Department of Pathology, Creighton University, Omaha, NE, USA; ^5^Department of Anesthesia, Mayo Clinic, Rochester, MN, USA

## Abstract

Docetaxel is a commonly used chemotherapeutic agent in a variety of cancer treatment regimens. We present a case of apparent docetaxel-induced Stevens-Johnson syndrome (SJS) in a patient recently treated for metastatic prostate cancer. This medication is not classically associated with the development of SJS but in our case, along with a number of other case reports, and a single phase II clinical trial, an association was recognized. We encourage clinicians who employ the use of this medication to be aware of this relationship.

## 1. Introduction

Stevens-Johnson syndrome (SJS) is a severe and potentially life-threatening skin condition that is associated with a number of medications and a few infectious agents. This condition, characterized by the separation of the epidermis from the underlying dermis, typically presents with a severe and painful rash that includes the conjunctiva and oral mucosa and is considered a dermatological emergency. Here, we present a case of apparent docetaxel-induced SJS, an association that has not been officially recognized.

## 2. Case Report

A 63-year-old male with a past medical history of hypertension, hyperlipidemia, and metastatic prostate adenocarcinoma to retroperitoneal lymph nodes, lung, and bone presented to the emergency room with a one-week history of a rash. The rash affected the hands, feet, back, and chest. It developed into blisters that later ruptured. The rash was especially painful in the hands and feet. He also reported red eyes and difficulty eating for the past week.

**Figure 1:**
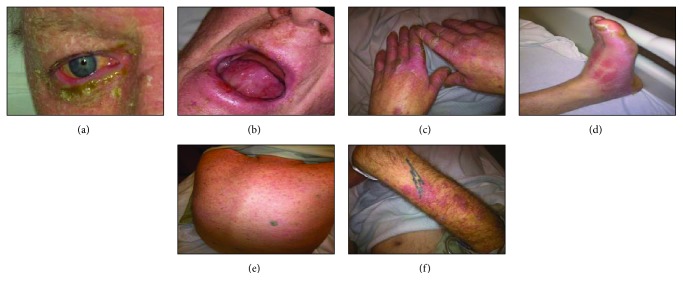
Patient images. Physical examination showed conjunctival, mucosal, and skin involvement. Stevens-Johnson syndrome affects less than 30% of the body surface area and commonly affects mucous membranes. The photographs presented in Figure 2 were obtained with patient permission and depict the lesions throughout his body. (a) Involvement of the conjunctiva of the right eye. (b) Mucosal ulcers of the oropharynx. (c–f) Other areas of skin involvement that totaled to less than 30% of total body surface.

Vital signs upon presentation were as follows: temperature 36.4°C, pulse 83/min, respiratory rate 12/min, and blood pressure 121/60 mmHg. Physical examination of the patient revealed a severe rash covering less than thirty percent of the body, oral ulcers, and conjunctival redness (Figure 1). Laboratory data at the time of admission is notable for leukocytosis, electrolyte imbalances, and hepatic dysfunction (Table 1).

The patient's current cancer treatment regimen included active hormonal therapy with leuprolide and active chemotherapy with docetaxel. He had received two cycles of docetaxel therapy (generic form: 75 mg/m^2^), with the last dose of docetaxel received two weeks prior to presentation. He was not on other medications at the time of admission.

Treatment of the patient included intravenous fluid replacement, prednisone, piperacillin/tazobactam, ondansetron, and morphine. Wound and eye care were provided. Dermatology was consulted.

**Figure 2:**
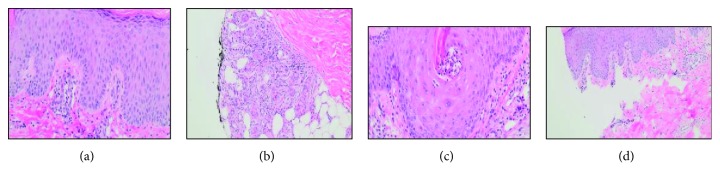
Biopsy images and pathology report. Microscopic examination of the skin (hematoxylin and eosin stain) was performed. The punch biopsies from the edges of both lesions (left forearm and left medial foot) show similar morphologic features. There is a predominantly normal intact stratum corneum overlying the epidermis. Within the dermis, there is interface dermatitis with a superficial perivascular infiltrate of lymphocytes with occasional neutrophils (a). Additionally, basal vacuolization with dyskeratotic keratinocytes scattered throughout all levels of the epidermis is seen. Mild interstitial and periadnexal lymphocytic inflammation are noted (b) as well as a focal area of neutrophils present in the keratin plug of a hair follicle (c). There is a focal area of incipient epidermal detachment present (d). No fungal elements or eosinophilic infiltration are noted in the specimens. The histologic features are consistent with toxic epidermal necrolysis/Stevens-Johnson syndrome.

Because our searches for an established association between SJS and docetaxel were in vain, we elected to obtain a punch biopsy of the lesions to establish pathological evidence of our diagnosis. The punch biopsies were obtained from the edges of lesions on the left forearm and left medial foot. Light microscopy of the hematoxylin and eosin-stained specimens were confirmatory for SJS (Figure 2).

The patient clinically improved with supportive therapy and was discharged home. He was scheduled a follow-up with his oncologist to discuss other treatment options.

## 3. Discussion

Docetaxel is an antitubulin agent that inhibits mitosis by stabilizing microtubule assembly. It is a widely used chemotherapeutic agent in the treatment of breast, lung, prostate, and other cancers [1]. The classically known side effects of docetaxel therapy include alopecia, pancytopenia, drug-induced liver injury, nausea, vomiting, and diarrhea. There have been an increasing number of generic docetaxel toxicities in comparison to the original drug formulation. Impurities, excipients, and the amount of active agent itself can all have an impact on the efficacy of the drug and impact its narrow therapeutic index resulting in increased side effects [2].

A number of popular clinical pharmacology resources do not include Stevens-Johnson syndrome (SJS) as a known complication of docetaxel chemotherapy. However, the current case and the cases written by a handful of other clinicians may provide clinical evidence that docetaxel therapy is associated with the development of this potentially life-threatening dermatologic condition.

Stevens-Johnson syndrome is one of the life-threatening dermatologic diseases, which is characterized by skin and mucosal involvement and systemic symptoms. Most often, SJS is secondary to medication use but it also could be related to an infection [3]. The treatment of this condition remains controversial but all reach agreement that supportive management is first line and the use of steroids, IVIG, tumor necrosis factor, or even cyclosporine lacks powered data. The mortality rate of SJS in severe cases could exceed 90 percent [4].

The existing reported cases of SJS-like skin reactions in association with docetaxel are few (Table 2). Dourakis et al. [5] described a case of a 60-year-old woman that received docetaxel as salvage chemotherapy for non-Hodgkin's lymphoma who developed toxic epidermal necrolysis. Similarly, Arshad et al. [6] shared a case of a 40-year-old woman receiving docetaxel for invasive ductal breast carcinoma who developed toxic epidermal necrolysis. Despite aggressive supportive measures, the patient died. Sawada et al. [7] reported the development of SJS in a patient with metastatic breast cancer after a single dose. This case also provided pathological evidence of SJS and ruled out conditions such as pemphigus vulgaris. Following the seventh dose of docetaxel in another patient with metastatic breast cancer, Moisidis and Möbus [1] encountered skin lesions consistent with erythema multiforme and SJS. Ohlmann et al. [8] reported the course of a patient receiving docetaxel who developed SJS and acute hepatic failure and died 6 weeks later. Kattan et al. [9] carried out a phase II trial to establish the safety of the combination of docetaxel, zoledronic acid, and estramustine in patients with metastatic prostate carcinoma. Of the 27 patients included in the trial, one patient died of SJS with liver failure.

An interesting point of discussion relative to this case is, first, the finding of elevated liver function tests. Our patient did not have a history of hepatic disease. However, docetaxel is known to be associated with hepatotoxicity [10]. This association may be relevant because a number of case reports summarized by Devarbhavi et al. [11] reported that patients with drug-induced liver dysfunction and SJS have poorer outcomes. These findings were especially prevalent in patients taking antiepileptics, antiretrovirals, and sulfonamides. Although it is unknown whether liver dysfunction predisposes patients to SJS, there appears to be an association that should be noted by physicians.

## 4. Conclusion

We recognize that a number of other authors have encountered apparent docetaxel-induced SJS and our report adds to the growing body of knowledge of this detrimental relationship. We encourage clinicians that employ the use of this medication to be cognizant of the risk of SJS and to treat this condition early and aggressively.

## Figures and Tables

**Table 1:** Laboratory data at the time of admission. Notable abnormalities include leukocytosis, electrolyte imbalances, and hepatic dysfunction.

Laboratory test	Value	Reference range
White blood cell count (k/*μ*l)	28.3	4.0-12.0
Hemoglobin (gm/dl)	12.0	13.5-17.5
Platelet (k/*μ*l)	176	140-440
Sodium (mmol/l)	130	135-145
Potassium (mmol/l)	3.3	3.7-5.1
Chloride (mmol/l)	97	96-110
Carbon dioxide (mmol/l)	26	22.0-32.0
Blood urea nitrogen (mg/dl)	15	6-24
Creatinine (mg/dl)	0.8	0.60-1.30
Glucose (mg/dl)	112	70-100
Calcium (mg/dl)	7.9	8.5-10.5
Protein (gm/dl)	5.8	6.0-8.4
Albumin (gm/dl)	2.6	3.5-5.0
Bilirubin (mg/dl)	3.6	0.0-1.5
Alkaline phosphatase (u/l)	323	33-138
Aspartate aminotransferase (u/l)	156	10-40
Alanine aminotransferase (u/l)	48	12-78

**Table 2:** Reported cases of docetaxel-induced SJS.

Author	Year	Type of article	Description of case/study	Outcome
Moisidis and Möbus [1]	2005	Case report	76-year-old female with metastatic breast cancer with docetaxel-induced erythema multiforme	Resolution of symptoms after 3 weeks following treatment with high-dose steroids
Dourakis et al. [5]	2002	Case report	60-year-old patient with non-Hodgkin's lymphoma and received salvage therapy with docetaxel and prednisone developed SJS within 5 days of treatment	Received treatment with supportive measures and survived
Arshad et al. [6]	2014	Case report	46-year-old female with metastatic breast cancer developed SJS two weeks following treatment with three cycles of docetaxel.	She was supportively managed.
Sawada et al. [7]	2009	Case report	56-year-old female treated for metastatic breast cancer with first cycle of docetaxel developed SJS.	She received treatment with topical clobetasol and triamcinolone and other supportive measures. The patient survived with resolution of symptoms.
Ohlmann et al. [8]	2007	Case report	67-year-old male with prostate cancer received docetaxel as a part of a randomized phase III trial developed SJS after five cycles of treatment.	Patient received systemic steroids and antibiotics for treatment and succumbed as a result of SJS secondary to docetaxel.
Kattan et al. [9]	2008	Clinical trial, open-labelled phase II	27 patients received weekly docetaxel and zoledronic acid and estramustine between 2002 and 2014 for hormone refractory prostate cancer.	1 patient died of docetaxel toxicity in the form of SJS, 2 weeks after the second cycle.
